# Tackling matrix effects in biosensor-based analysis of untreated blood plasma

**DOI:** 10.1007/s00216-026-06312-9

**Published:** 2026-01-19

**Authors:** Giusy Finocchiaro, Markéta Bocková, Jiří Homola

**Affiliations:** Institute of Photonics and Electronics of the Czech Academy of Sciences, Chaberská 1014/57, 182 00 Prague, Czech Republic

**Keywords:** Biosensor-based analysis, Surface plasmon resonance, Matrix effects, Human blood plasma

## Abstract

Human blood plasma is the matrix of choice for clinical diagnostic applications. However, matrix effects associated with its complex composition interfere with the accurate detection of biomarkers that are often present at extremely low concentrations. This study investigates such matrix effects, particularly the nonspecific adsorption of blood plasma to the sensor surface and the interaction of endogenous antibodies with the used receptors, and shows how they can be discriminated using a surface plasmon resonance biosensor. Moreover, we describe a strategy to tackle matrix effects by combining sequential blood plasma/buffer injections, single-surface referencing, and the addition of an antibody against endogenous immunoglobulins to blood plasma. Finally, we apply this strategy to the detection of the cancer biomarker carcinoembryonic antigen in undiluted blood plasma, achieving detection limits of 12 ng/mL using direct detection, and 225 pg/mL using a sandwich assay with functional gold nanoparticles.

**Supplementary Information:**

The online version contains supplementary material available at https://doi.org/10.1007/s00216-026-06312-9.

## Introduction

Human blood plasma is a complex biological matrix that contains a broad spectrum of biomarkers and represents the medium of choice for clinical diagnostics. However, the most abundant blood plasma components, which are present at levels vastly exceeding those of biomarkers, produce matrix effects that interfere with analytical measurements [1]. Matrix effects represent a major concern not only for conventional analytical methods and immunoassays, such as enzyme-linked immunosorbent assays (ELISA), but also for biosensing technologies, such as surface plasmon resonance (SPR) biosensors [2, 3]. The matrix effects that impair the antibody-based detection of protein biomarkers with SPR biosensors include (i) the fouling or nonspecific adsorption (NSA) of blood plasma components to the sensor surface [1] and (ii) the interference of endogenous antibodies (hereafter referred to as interfering antibodies, IAbs) with the used receptors [4, 5].

Various approaches have been proposed to suppress the effects of NSA in SPR biosensors, such as sample treatment (i.e., dilution, filtration, or depletion of the most abundant components) [1, 6], functionalization of biosensors [7–10] and functional nanoparticles [11–13] with low-fouling coatings, and advanced detection methodologies and assay schemes [6, 14–16]. The latter includes the use of referencing approaches to compensate for the NSA contribution to the sensor response, such as the single-surface referencing (SSR) method [15], and the implementation of microfluidic approaches for washing weakly adsorbed components from the sensor surface, such as the sequential injection method [16]. However, none of these approaches address the interfering effects associated with the interaction of the IAbs with the receptors, which are typically neglected or improperly referred to as NSA in most biosensor-based analytical studies [2, 6]. Moreover, although a few investigations have reported biomarker detection in untreated biological matrices [17–21], the majority of these approaches have been demonstrated to work well only for biomarker detection in diluted biological matrices.

In this study, we show how the NSA and IAb interference can be discriminated using an SPR biosensor. We combine the SSR and sequential injection approaches to mitigate the effects of NSA, and the use of an antibody against immunoglobulin G (Ab_IgG_) to minimize the effects associated with the IAbs. Applying this strategy, we demonstrate the detection of carcinoembryonic antigen (CEA), a biomarker associated with the progression of multiple cancer types [22], in untreated blood plasma samples.

## Materials and methods

### Blood plasma

Human blood samples were collected at the Institute of Hematology and Blood Transfusion (Prague, Czech Republic) in compliance with the ethical requirements of the Declaration of Helsinki. The blood plasma was separated from human blood by centrifugation at 460 × *g* for 10 min. Ethylenediaminetetraacetic acid (EDTA) was added as an anticoagulant, and the samples were stored in EDTA-coated tubes at − 80 °C until use. To obtain a representative blood plasma sample [23], we used a pooled sample derived from a cohort of ten healthy individuals of mixed genders.

### SPR biosensor and chip functionalization

The six-channel SPR biosensor with temperature stabilization used in this work was developed at the Institute of Photonics and Electronics (Prague, Czech Republic) [16]. The SPR platform is based on the Kretschmann geometry and spectral modulation. In this geometry, a polychromatic light is directed onto the SPR chip at a fixed angle. The excitation of surface plasmons generates a characteristic dip in the spectrum of reflected light. A change in the refractive index due to the binding of biomolecules to the SPR chip produces a shift in the excitation wavelength, which is used as the sensor response. For the developed SPR platform, a sensor response (wavelength change) of 1 nm corresponds to a protein surface density of 17 ng/cm^2^ (for details, see Supplementary information, SI).

The SPR chip was prepared by depositing 1.5 nm of titanium and 50 nm of gold on a glass substrate using electron-beam evaporation in vacuum. Prior to each biosensing experiment, the SPR chip was incubated overnight with a mixture of carboxy- and hydroxy-terminated alkanethiols at a total concentration of 200 μM in a 3:7 molar ratio. The carboxylic groups were activated on the SPR platform using 25 mM N-hydroxysuccinimide (NHS) and 95.3 mM 1-ethyl-3-(3-dimethylaminopropyl)-carbodiimide hydrochloride (EDC) for 10 min at a flow rate of 5 µL/min. Then, the primary antibody against CEA (Ab_1,CEA_) was diluted in 10 mM sodium acetate (SA_10_) to a final concentration of 10 μg/mL and flowed over the SPR chip at 20 μL/min to saturation, followed by a 2-min washing with high ionic strength phosphate buffer (PBS_NaCl_). The surface was then passivated with 100 µg/mL bovine serum albumin (BSA) in SA_10_ at 20 µL/min to saturation, followed by PBS_NaCl_ and 0.5 M ethanolamine injections for 5 min. All buffers and reagents, along with their suppliers, are reported in Tables S1 and S2 in SI.

### Approach

Herein, we combined the sequential blood plasma injection approach with the SSR method. The sequential injection approach involves alternating injections of blood plasma and a high ionic strength buffer to wash weakly adsorbed components from the sensor surface [16]. We used a dispersionless microfluidic system (Fig. S1a) with two peristaltic pumps and PBS_NaCl_ with 250 µg/mL BSA (PBS_NaCl_ _+ BSA_) as the washing buffer. The blood plasma sample was injected eleven times for 30 s, each injection followed by a washing step with a duration of 30 s, both performed at a flow rate of 20 μL/min. The sensorgrams illustrating the sequential injection approach are provided in Figs. S1b and S1c; the sensorgrams in Fig. S2 demonstrate that the used washing buffer did not disrupt the specific CEA–Ab_1,CEA_ interaction.

The SSR approach implies compensating for the NSA contribution to the blood plasma sensor response by using a reference channel that presents the same surface functionalization as the detection channel [15]. Specifically, a three-channel configuration was employed, involving one detection channel (spiked with a defined concentration of CEA) and two reference channels (bare blood plasma and blood plasma spiked with Ab_1,CEA_). Comparison of these channels enabled the determination of the natural, spiked, and total CEA concentrations, as well as the assessment of the influence of endogenous antibodies. No treatment or dilution of the blood plasma sample was performed, except for the addition of the above-mentioned reagents (i.e., buffer with spiked CEA, Ab_1,CEA_, or Ab_IgG_), which constituted only 3% of the total sample volume.

### Experimental

To establish the calibration curves for the detection of CEA in blood plasma, we measured the reference-compensated sensor response to concentrations of 50, 100, 200, 300, 500, and 1000 ng/mL CEA spiked in blood plasma. In addition to the direct detection, we performed a sandwich assay with a biotinylated secondary antibody against CEA (b-Ab_2,CEA_) and streptavidin-coated gold nanoparticles (AuNPs, see SI for their preparation). The b-Ab_2,CEA_ was diluted to a concentration of 10 µg/mL in phosphate buffer supplemented with 0.01% Tween 20 (PBS_Tween_) and injected for 15 min at 10 μL/min. The AuNPs were diluted in PBS_Tween_ to a concentration of 25 pM and flowed over the sensor surface for 60 min at 5 μL/min. The same protocol was used to establish the calibration curves for the detection of CEA in blood plasma with added Ab_IgG_ (10 µg/mL). In these experiments, we measured the reference-compensated sensor response to 0.3, 1, 3, 6, 10, 15, 20, 50, 100, 200, 300, 500, and 1000 ng/mL CEA spiked in blood plasma, both with and without the enhancement due to AuNPs. The calibration curves for the direct detection of CEA were created by plotting the reference-compensated sensor response to blood plasma taken 10 min after the last buffer injection. The calibration curves for the AuNP-enhanced detection of CEA were created by plotting the reference-compensated sensor response to the AuNPs taken 5 min after the buffer injection. For comparison, the level of naturally occurring CEA in blood plasma was measured using a commercial ELISA kit from Abbott Diagnostics, USA.

## Results and discussion

**Fig. 1:**
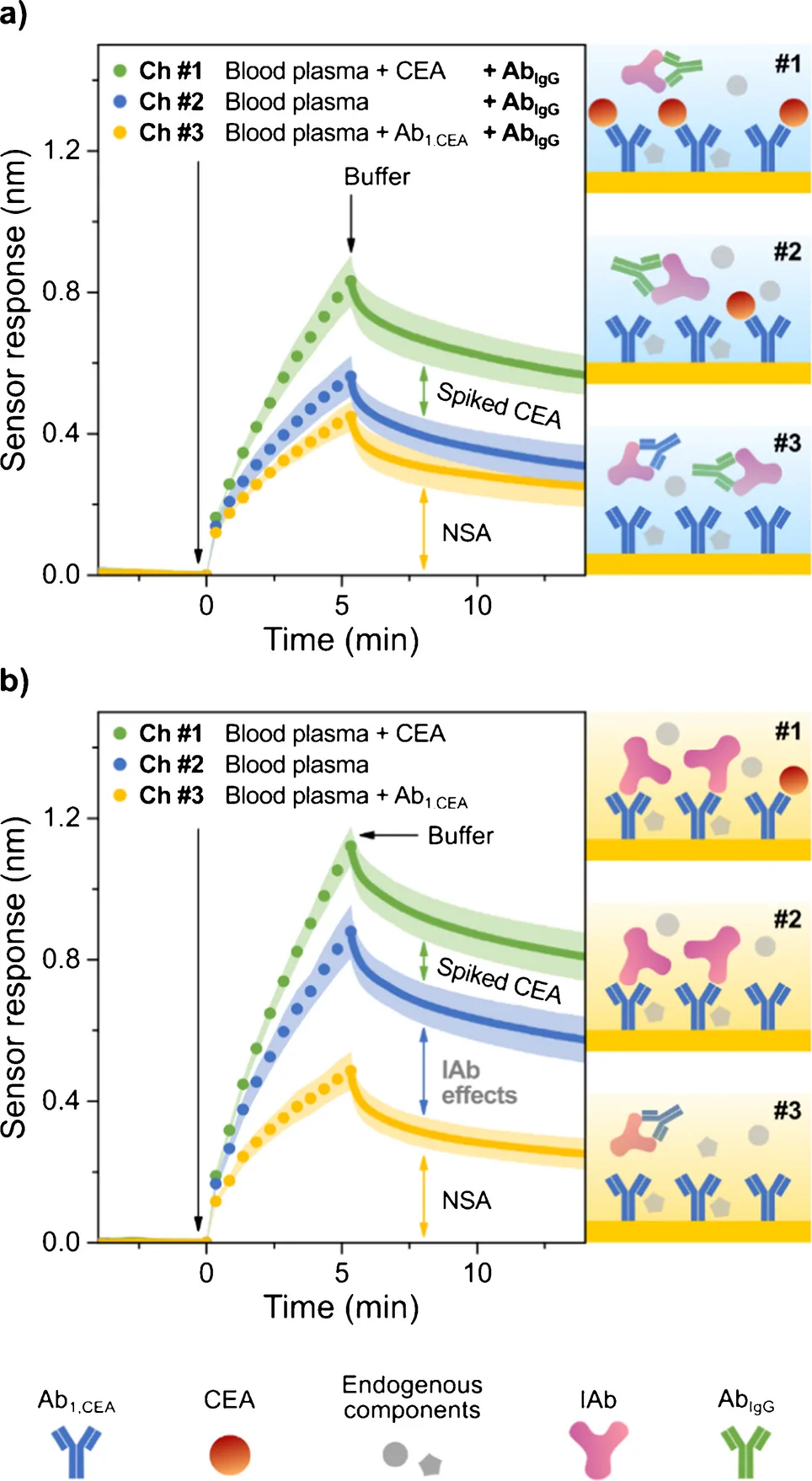
Average sensor response to the binding of blood plasma spiked with 300 ng/mL CEA (Ch #1), blood plasma alone (Ch #2), and blood plasma spiked with Ab_1,CEA_ (Ch #3), after adding Ab_IgG_ to the blood plasma sample (**a**), and without Ab_IgG_ addition (**b**). The shaded areas correspond to the standard deviations of at least three measurements. The panels on the right side illustrate the interactions occurring in Ch #1, Ch #2, and Ch #3 (the legend of symbols is displayed in the panel below)

The approach used for CEA detection is illustrated in Fig. 1a and b, showing the sensor response to undiluted blood plasma with and without Ab_IgG_ addition. In both scenarios, Ch #1 provides the sensor response to blood plasma spiked with 300 ng/mL CEA, Ch #2 provides the sensor response to blood plasma, and Ch #3 (reference channel) provides the sensor response to blood plasma spiked with Ab_1,CEA_. Figure 1a reveals a minor difference in sensor response between Ch #2 and Ch #3, which may be attributed to the naturally occurring CEA in blood plasma; whereas Fig. 1b shows a substantial difference between Ch #2 and Ch #3, suggesting a strong interfering effect associated with endogenous IAbs. Overall, Fig. 1a and b indicate that the addition of Ab_IgG_ to blood plasma significantly reduced the influence of IAbs (sensor response difference between Ch #2 and Ch #3), while it had a negligible impact on the NSA (Ch #3) and on the specific binding of CEA spiked in blood plasma (sensor response difference between Ch #1 and Ch #2). To further examine the effect of IAbs, we performed an SPR experiment using the same blood plasma sample that was depleted of the most abundant proteins (i.e., human serum albumin and IgG). In this experiment, only a negligible sensor response difference between Ch #2 and Ch #3 was observed following depletion (Fig. S3), suggesting that the sensor response to untreated blood plasma was mainly due to endogenous abundant proteins rather than naturally occurring CEA. The role of Ab_IgG_ was further explored in experiments using the SPR biosensor for the detection of two other analytes, human chorionic gonadotropin and horseradish peroxidase (Fig. S4), as well as for the detection of CEA in another blood plasma sample (Fig. S5). The results confirmed that adding Ab_IgG_ to blood plasma (independently of the analyte and sample) reduced the sensor response difference between Ch #2 and Ch #3, and thus, the effects associated with IAbs.

**Fig. 2:**
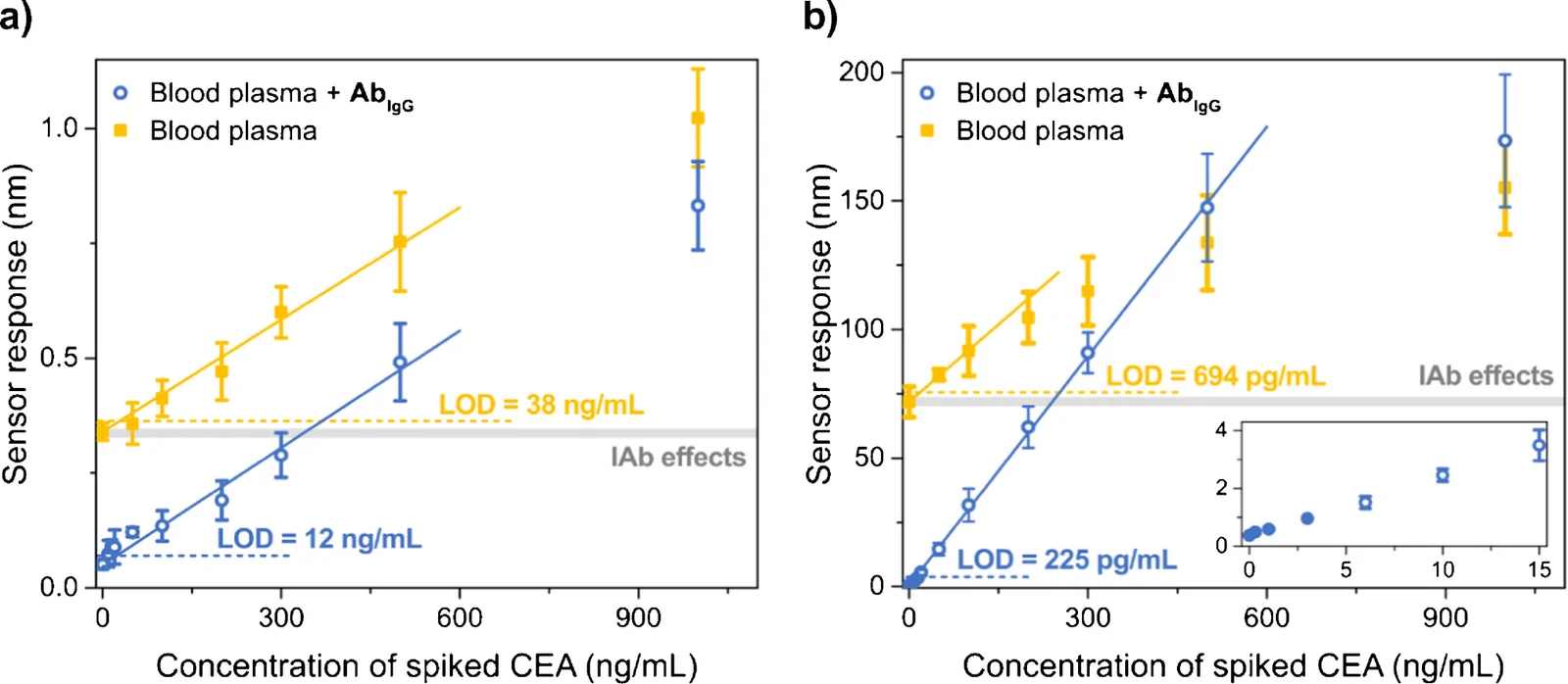
Calibration curves for the detection of CEA in blood plasma with and without Ab_IgG_ addition (blue and yellow scatter, respectively), using direct detection (**a**) and a sandwich assay with AuNPs (**b**). The solid lines indicate the linear fits of the data points, and the error bars represent the standard deviations of at least three measurements. The inset in **b** shows the reference-compensated sensor response to low CEA concentrations spiked in blood plasma

We measured the sensor response as a function of CEA concentration with and without Ab_IgG_ addition to blood plasma for both direct detection and AuNP-enhanced detection (Fig. 2a and b). Each data point represents the average of at least three independent measurements, with the error bars indicating the standard deviations of the individual measurements. The standard deviation typically fell within 10–15% of the sensor response. The offsets of the calibration curves for CEA detection in blood plasma without the addition of Ab_IgG_ (gray horizontal lines in Fig. 2a and b) were mainly associated with the interaction of IAbs with Ab_1,CEA_ immobilized on the sensor surface. The sensor response to the limit of detection (LOD) was calculated from the standard deviation of at least 13 replicates of the blank measurement (Ch #3), by applying the Student’s *t*-distribution with a confidence interval of 97.5% (for details, see SI). The resulting LODs suggest that the addition of Ab_IgG_ to blood plasma improves the LOD for CEA direct detection by a factor of ~ 3, while reducing the offset of the calibration curve by approximately one order of magnitude (Table S3 in SI). The achieved LOD of 12 ng/mL represents an improvement over previously reported LOD values for CEA direct detection with SPR, namely 30 ng/mL in 50% blood plasma [24] and 76 ng/mL in undiluted blood serum [18]. To enable the detection of CEA in untreated blood plasma at physiological concentrations (i.e., below 3 ng/mL [22]), a sandwich assay with AuNPs was performed (Fig. 2b). While the calibration curve for blood plasma with the addition of Ab_IgG_ starts approximately at zero for the sample not spiked with CEA, the calibration curve obtained when no Ab_IgG_ was added to blood plasma is offset by about 72 nm. These results indicate that the functional AuNPs were also binding to IAbs captured at the sensor surface. The sensor response was enhanced by the AuNPs by a factor of about 300 and 210 for blood plasma with and without Ab_IgG_ addition (estimated from the linear ranges of the calibration curves). The LODs determined for CEA detection in blood plasma with and without Ab_IgG_ were 225 pg/mL and 694 pg/mL, respectively. The LOD obtained with the presented methodology (225 pg/mL) is tenfold below the physiological threshold and is comparable to previously reported LODs for AuNP-enhanced SPR-based detection in 50% blood plasma [12, 24, 25] or in 0.1% blood serum [26]. It should be noted that the offset of 0.37 nm observed at zero concentration of CEA in blood plasma after Ab_IgG_ addition (inset in Fig. 2b) corresponds to a concentration of naturally occurring CEA of 1.2 ng/mL, which was in excellent agreement with the value determined by ELISA (1.7 ng/mL). The presented strategy enabled the detection of CEA over a dynamic range spanning 3 orders of magnitude, from the lowest measured concentration of 0.3 ng/mL to the concentration of 500 ng/mL CEA, which approaches the highest CEA concentrations observed at pathological conditions [27].

## Conclusions

We investigate and discriminate between blood plasma matrix effects, particularly the nonspecific adsorption to the sensor surface and the interaction of endogenous antibodies (IAbs) with the receptor, using an SPR biosensor. We combine the sequential injection and single-surface referencing approaches to mitigate the nonspecific adsorption, along with Ab_IgG_ to reduce the effects associated with IAbs. We apply this strategy to the detection of CEA in undiluted blood plasma using an SPR biosensor, demonstrating a reduction of IAb effects by a factor of 10 when using direct detection and nearly 200 when using a sandwich assay with AuNP enhancement. The approach enables CEA direct detection in undiluted blood plasma at levels as low as 12 ng/mL, representing the best LOD achieved by an SPR biosensor for CEA direct detection in blood plasma or serum. In conjunction with the sandwich assay, the proposed strategy yields an LOD of 225 pg/mL, which is an order of magnitude below the physiological threshold. This suggests that the proposed methodology enables the robust detection of clinically relevant levels of CEA in untreated blood plasma samples. This approach can potentially be adapted to a broad range of protein biomarkers and biological matrices. The benefits are expected to depend on the composition of the biological matrix, particularly on the concentration of IAbs; higher performance improvement may be expected in matrices with higher IAb levels.

## Supplementary Information

Below is the link to the electronic supplementary material.

Supplementary file1 (PDF 608 KB)

## Data Availability

The data are available at the link https://doi.org/10.5281/zenodo.17340883.
