# Supplementary material for: Tackling matrix effects in biosensor-based analysis of untreated blood plasma
Source: Anal Bioanal Chem. 2026 Jan 19;418(23):7839–44. doi: 10.1007/s00216-026-06312-9 (PMC13744519; doi:10.1007/s00216-026-06312-9)
Supplement: Supplementary file 1 [file 216_2026_6312_MOESM1_ESM.pdf]

Supplementary Information

Tackling matrix effects in biosensor-based analysis of untreated blood plasma

Giusy Finocchiaro, Markéta Bocková, Jiří Homola*

Institute of Photonics and Electronics of the Czech Academy of Sciences, Chaberská 1014/57, 182 00 Prague, Czech Republic

Corresponding author: Jiří Homola, e-mail address: homola@ufe.cz

Contents

S1. Materials and methods 2

S1.1. Protein surface density calculation 2

S1.2. Buffers and reagents 2

S1.3. Functional gold nanoparticles 3

S2. Dispersionless microfluidic system and sequential injection approach 4

S3. Supplementary results 5

S3.1. Effect of blood plasma depletion 5

S3.2. Effect of Ab_{IgG} addition to blood plasma 6

S4. LOD determination 7

S5. References 8

S1. Materials and methods

S1.1. Protein surface density calculation

The surface density of proteins was determined using the *de Feijter* formula below [1, 2]:

$$\Delta n = \left(\frac{dn}{dc}\right)_{vol} \frac{\Delta \Gamma}{h} ,$$

where Δn is the refractive index change associated with the protein binding to the sensor surface, $(dn/dc)_{vol}$ is the volume refractive index increment [3], $\Delta \Gamma$ is the protein surface density, and h is the thickness of the adsorbed layer of proteins. The surface refractive index change (Δn) is obtained by dividing the sensor response ($\Delta \lambda$) by the surface refractive index sensitivity (S_s), yielding the following equation for $\Delta \Gamma$:

$$\Delta \Gamma = \frac{\Delta \lambda}{S_s} \frac{h}{\left(\frac{dn}{dc}\right)_{vol}} .$$

S1.2. Buffers and reagents

Table S1. List of (i) buffers, (ii) chemicals, and (iii) proteins used in the surface plasmon resonance (SPR) experiments and gold nanoparticle preparation, including acronym, composition, pH (for buffers), supplier, and catalogue number (Cat. No.). All buffers were prepared by dilution in deionized water (Q-water, 18 MΩ/cm resistivity, Direct-Q UV3, from Millipore, USA).

(i) Buffers

Name	Acronym	Composition	pH	Supplier	Cat. No.
Sodium acetate	SA_{10}	10 mM SA	5	Sigma-Aldrich	57899
Phosphate buffer	PBS	1.4 mM KH_2PO_4, 8 mM Na_2HPO_4, 2.7 mM KCl, and 137 mM NaCl	7.4	Sigma-Aldrich	P5368
High ionic strength phosphate buffer	PBS_{NaCl}	1.4 mM KH_2PO_4, 8 mM Na_2HPO_4,<br>7 mM KCl, and 750 mM NaCl	7.4	Sigma-Aldrich<br>Penta	60218<br>71649<br>16210-31000<br>16610-31000
3-(N-morpholino) propanesulfonic acid	MOPS	1 mM MOPS	7.4	Sigma-Aldrich	M1254

(ii) Chemicals

Name	Acronym / Formula	Supplier	Cat. No.
11-mercapto-hexa(ethylenglycol)undecyloxy acetic acid	Carboxy-terminated alkanethiol	Prochimia	TH003m11n6-0.2
11-mercapto-tetra(ethylenglycol)undecanol	Hydroxy-terminated alkanethiol	Prochimia	TH001m11n4-0.2
N-hydroxysuccinimide	NHS	Cytiva	
1-ethyl-3-(3-dimethylaminopropyl)-carbodiimide hydrochloride	EDC	Cytiva	14100050
Ethanolamine hydrochloride	EA	Cytiva	
Tetrachloroauric (III) acid trihydrate	$HAuCl_4 \cdot 3H_2O$	Sigma-Aldrich	520918

Trisodium citrate	$C_6H_5Na_3O_7$	Sigma-Aldrich	C8532
Sodium hydroxide	NaOH	Penta	15760-31000
Polysorbate 20	Tween 20	Sigma-Aldrich	P9416

(iii) Proteins

**Name**	**Acronym**	**Supplier**	**Cat. No.**
Carcinoembryonic antigen	CEA	Biosynth	30-1819
Bovine serum albumin	BSA	Sigma-Aldrich	A3059
Streptavidin	S	Sigma-Aldrich	S4762

Table S2. List of antibodies used in the SPR experiments, including their clonality, host, target, role, type, supplier, and catalogue number (Cat. No.). Abbreviations: Ab – antibody; CEA – carcinoembryonic antigen; IgG – immunoglobulin G; Fc – fragment crystallizable region; hCG – human chorionic gonadotropin; HRP – horseradish peroxidase; mc – monoclonal; ms – mouse; 1ary – primary antibody; 2ary – secondary antibody; BA – blocking agent.

**Acronym**	**Clonality**	**Host**	**Target**	**Role**	**Type**	**Supplier**	**Cat. No.**
$\mathbf{Ab_{1,CEA}}$	mc	ms	CEA (epitope Gold 1(*))	1ary	IgG1	Biosynth	10-C10F
$\mathbf{b\text{-}Ab_{2,CEA}}$	mc	ms	CEA (epitope Gold 4(*))	2ary	IgG1	Biosynth	61-C10GB
$\mathbf{Ab_{IgG}}$	mc	ms	Human IgG Fc (i.e., IgG1, 2, 3, and 4)	BA	IgG2a	Sigma-Aldrich	MAB1302
$\mathbf{Ab_{hCG}}$	mc	ms	hCG β-subunit	1ary	IgG1	Scripps	MC097
$\mathbf{Ab_{HRP}}$	mc	ms	HRP	1ary	IgG1	Biosynth	10R-1969

(*) The term Gold 1–5 denotes the epitopes of CEA [4]. Antibodies targeting epitope Gold 4 are the most commonly used in commercial enzyme-linked immunosorbent assays (ELISA), followed by antibodies targeting epitope Gold 1 [5, 6].

S1.3. Functional gold nanoparticles

Spherical gold nanoparticles (AuNPs) were synthesized using the seeded growth strategy [7, 8]. Briefly, a solution of 25 mM $HAuCl_4 \cdot 3H_2O$ was added to a boiling solution of 2.2 mM trisodium citrate to a final concentration of ~ 170 µM. The solution was cooled to 90 °C, followed by the addition of 1 mL of freshly prepared 60 mM trisodium citrate and 1 mL of 25 mM $HAuCl_4 \cdot 3H_2O$. These additions were repeated 3 times until the desired size of 50 nm was achieved (measured with a scanning electron microscope, e_Line Plus system from Raith, Germany; data not shown).
The synthesized 50 nm AuNPs were coated with streptavidin, following a procedure based on the protocol in Ref. [9]. Briefly, a dispersion of AuNPs at a concentration of ~ 1 nM (assessed with a NanoPhotometer, N60, from Implen, Germany) was incubated overnight with 333 µM carboxy-terminated alkanethiols. The obtained AuNPs were subsequently washed by adding an aqueous solution of 0.2 mM NaOH (Q_{NaOH}) and centrifuging at 10,000 × *g* for 10 min (using a High-Performance Centrifuge, Avanti J-E from Beckman Coulter, USA). The supernatant was discarded after centrifugation, and each washing step was repeated at least 7 times. The concentration of AuNPs was adjusted to ~ 2 nM, followed by the incubation with an aqueous mixture of 750 µM NHS and 570 µM EDC for 5 min, to activate the carboxylic groups, and with streptavidin for 1 hour, to obtain streptavidin-coated AuNPs. The streptavidin was diluted in MOPS buffer with 0.001% Tween 20 to a final concentration of 1 µM. Then, 0.1 M EA was added to the AuNPs to block the unreacted carboxylic groups, followed by the washing of the AuNPs with Q_{NaOH} (as reported above). Finally, the concentration of AuNPs was adjusted to ~ 1 nM; the solution containing AuNPs was stored at 4 °C until use.

S2. Dispersionless microfluidic system and sequential injection approach

The dispersionless microfluidic system used in this work is based on the exchange of liquid samples directly at the sensor surface, thereby reducing the effects of intermixing and slow fluid displacement [10]. In this design, each channel has two inlets (connected to two peristaltic pumps for sample injection) and two outlets (connected to microelectric valves and to waste). The microelectric valves allow opening one outlet at a time, which sets the flow direction of the two liquid samples. Unlike in conventional systems, in this design, the two liquids do not go through the same fluidic route and do not intermix (see Fig. S1a).

The use of the dispersionless microfluidics for biomolecular interaction analysis was demonstrated in Ref. [11], where the microfluidic system enabled the rapid and sequential injection of blood plasma and a washing buffer. The duration of the whole sequential injection procedure, including the washing steps, was 630 s, with an effective contact time of blood plasma with the sensor surface of 330 s. The 40-nm sensor response corresponding to the blood plasma steps (Fig. S1b) was due to the refractive index difference between the blood plasma sample and the washing buffer. The sensor response ~ 30 s after the injection of the washing buffer was used as the sensor readout (black scatter in Fig. S1b). The time axis was adjusted to account for the contact time of blood plasma with the sensor surface by removing the blood plasma steps from the sensorgram (Fig. S1c).

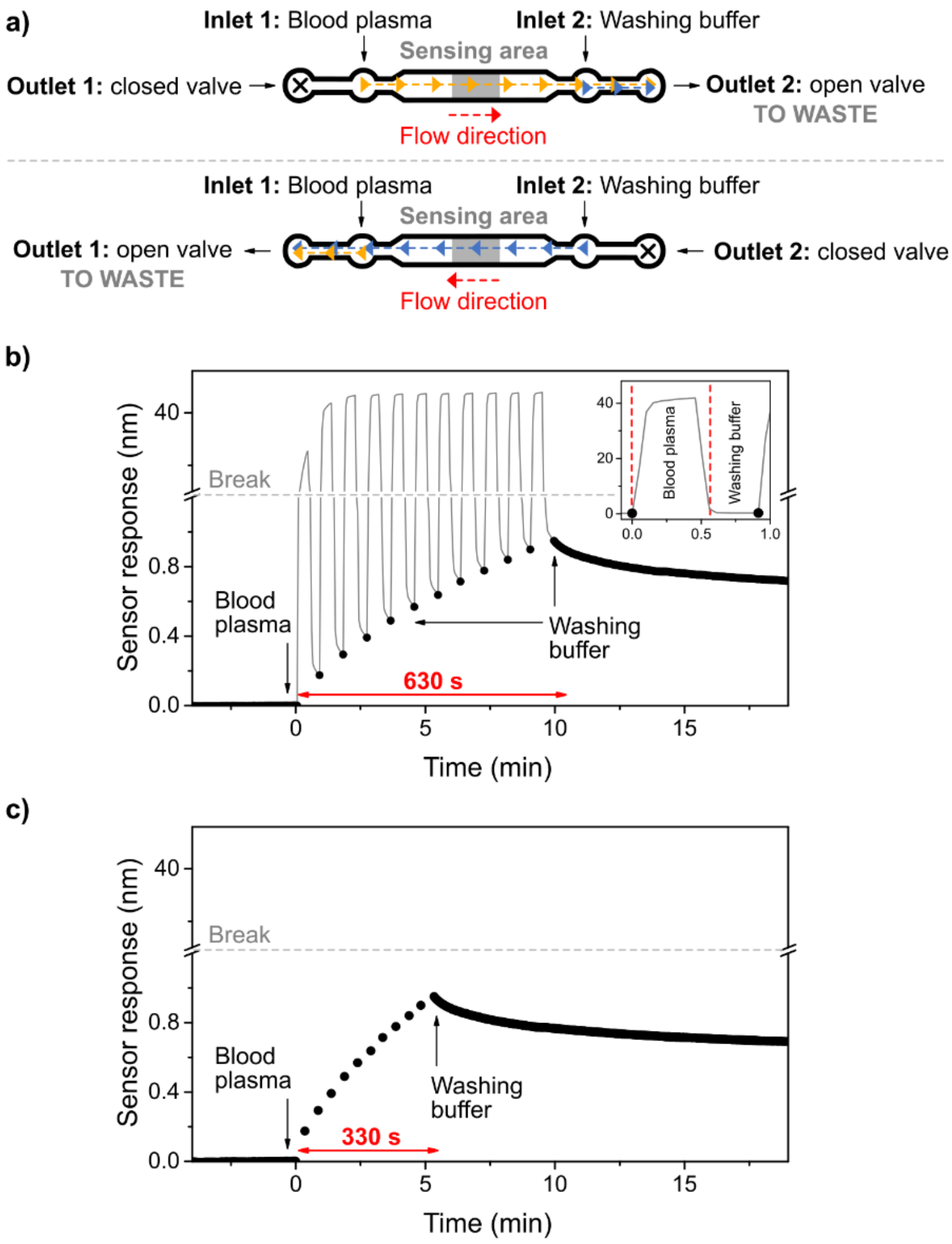

Fig. S1. (a) Principle of operation of the dispersionless microfluidic system. (b) Sensor response to the sequential injection of blood plasma and a washing buffer (inset with the magnification of a single blood plasma injection step followed by the washing buffer). (c) Processed sensor response.

Fig. S2 shows the SPR sensor response to 2 µg/mL CEA in $PBS_{NaCl + BSA}$ buffer using the sequential injection approach and the standard continuous injection of the sample through the flow cell. The close agreement between sensor responses indicates that neither the sequential injection procedure nor the washing buffer disrupted the specific CEA–$Ab_{1,CEA}$ interaction (Fig. S2).

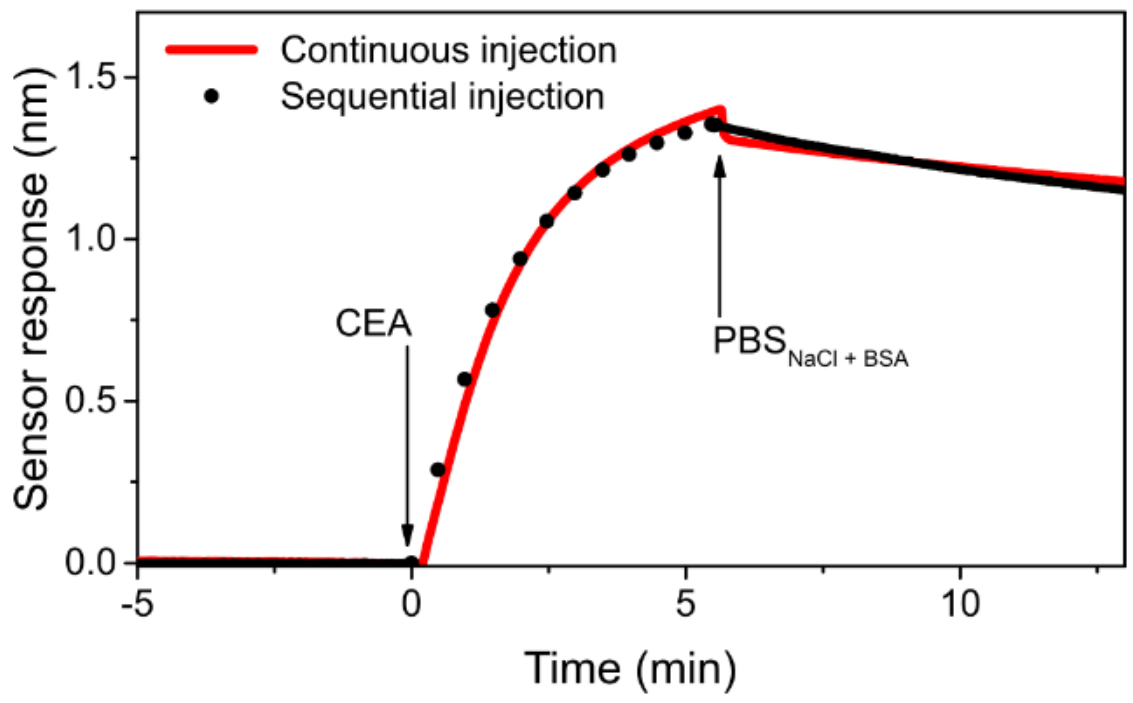

Fig. S2. Sensor response to 2 µg/mL CEA in $PBS_{NaCl + BSA}$ buffer injected using the continuous (red solid line) and sequential (black scatter) approaches.

S3. Supplementary results

S3.1. Effect of blood plasma depletion

To better understand the nature of the interference, we compared the sensor response to untreated blood plasma with that to blood plasma after depleting the most abundant proteins, including human serum albumin and IgG. Protein depletion was performed using a resin obtained from Thermo Fisher Scientific (*High Select*^{TM}). Fig. S3 shows that the blood plasma sample exhibited only a negligible sensor response following depletion.

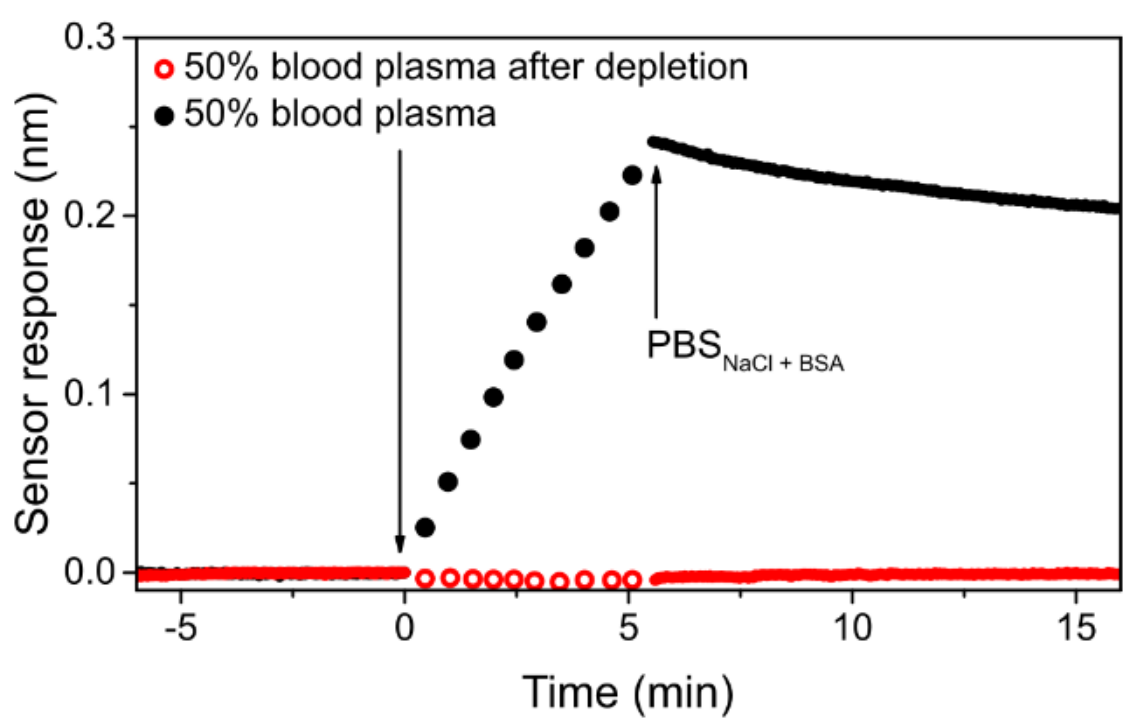

Fig. S3. Reference-compensated sensor response to the binding of depleted and non-depleted 50% blood plasma, flowed over a sensor surface functionalized with $Ab_{1,CEA}$.

S3.2. Effect of Ab_{IgG} addition to blood plasma

In addition to CEA, we investigated the effect of Ab_{IgG} on SPR-based immunoassays for the detection of two other analytes: human chorionic gonadotropin (hCG) and horseradish peroxidase (HRP). hCG is a biomarker of malignant trophoblastic disease (choriocarcinoma), and the immunoassays associated with its detection have been reported to be susceptible to interference from endogenous antibodies [12]. HRP is a non-human enzyme that has been used for assay development and sensing [13, 14]. The results in Fig. S4 show a reduction in the sensor response primarily associated with interfering antibodies (IAbs, grey columns) after the addition of Ab_{IgG} to blood plasma (red columns). This sensor response reduction was comparable between the SPR-based immunoassays for the detection of hCG and HRP.

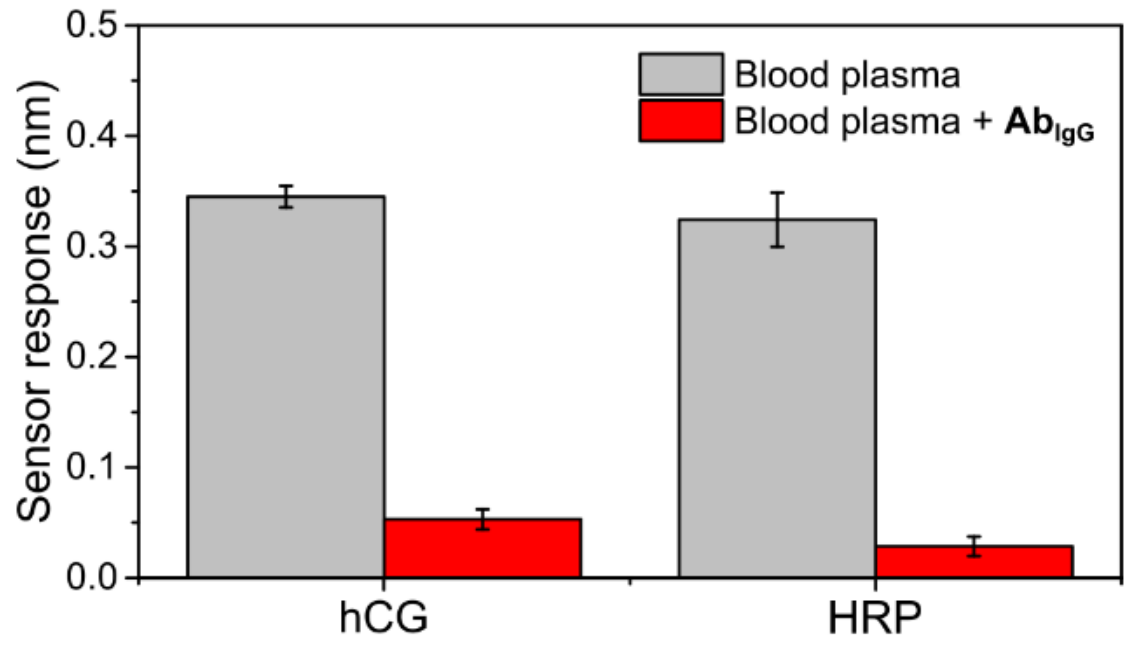

Fig. S4. Column bars corresponding to the reference-compensated sensor response to blood plasma flowed over two sensor surfaces functionalized with Ab_{hCG} and Ab_{HRP}, without and with Ab_{IgG} addition (grey and red bars, respectively).

We also examined the effect of blood plasma composition by applying the SPR-based immunoassay for CEA detection to an additional blood plasma sample pooled from a cohort of ten healthy donors. We observed a reduction in the sensor response due to the IAbs from ~ 0.02 nm to ~ 0 nm (Fig. S5).

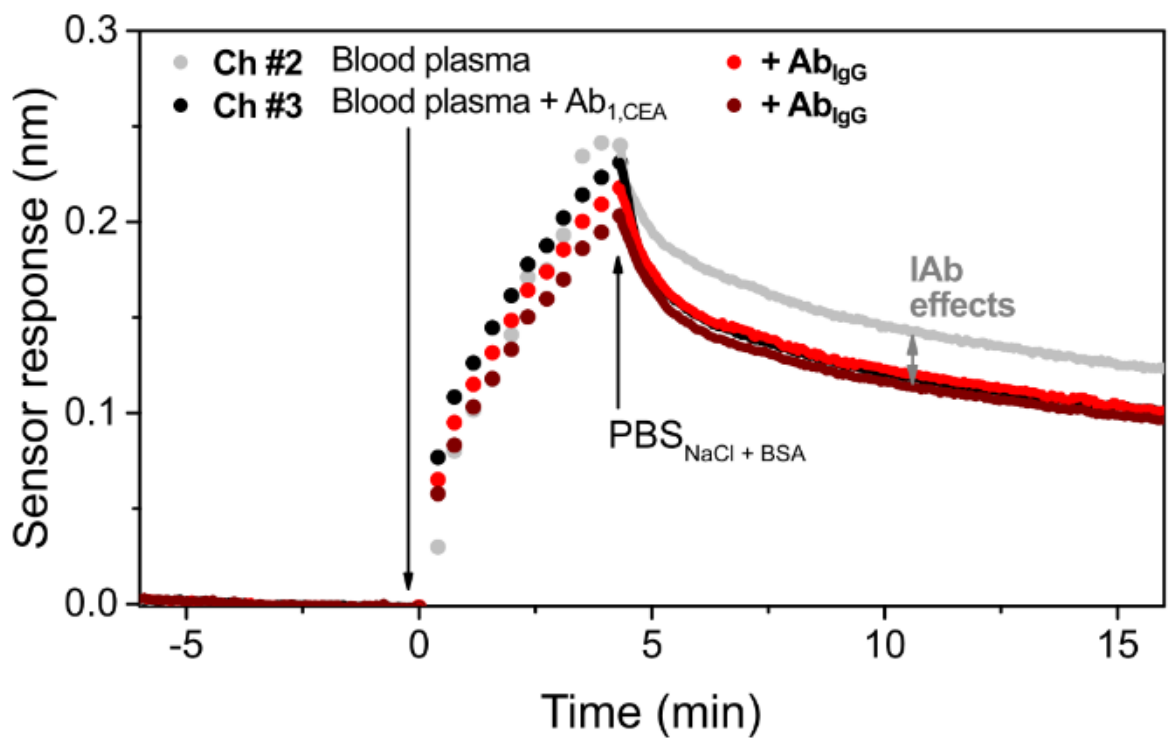

Fig. S5. Sensor response to the binding of blood plasma (Ch #2) and blood plasma spiked with $Ab_{1,CEA}$ (Ch #3), with and without Ab_{IgG} addition to blood plasma.

S4. LOD determination

The limit of detection (LOD) was calculated according to the International Union of Pure and Applied Chemistry (IUPAC) recommendations [15]. The LOD sensor response (R_{LOD}) was calculated as:

$$R_{LOD} = R_{ref} + t_{\alpha,n-1}\sigma \ ,$$

where R_{ref} is the reference-compensated sensor response to the blank sample (blood plasma spiked with $Ab_{1,CEA}$), t is the critical value of the Student's t-distribution, α is the significance level (equal to 0.025, corresponding to a confidence interval of 97.5%), n is the number of blank sample measurements (15 for the direct detection, 13 for the sandwich assay), and σ is the standard deviation of R_{ref}, also referred to as R_{ref} variability.

The LOD (the concentration corresponding to the R_{LOD}) was obtained by dividing R_{LOD} by the sensor sensitivity (S), determined as the slope of the calibration curve ($R = S{\cdot}c + o$). The offset of the calibration curve (o) in the absence of Ab_{IgG} was attributed mainly to IAb interaction with $Ab_{1,CEA}$.

Table S3. Parameters used for the determination of the LOD for (i) direct and (ii) AuNP-enhanced detection of CEA. Abbreviations: S – sensor sensitivity; o – offset of the calibration curve (associated with IAb interference); σ – variability of blank; n – number of performed measurements of blank; R_{LOD} – sensor response corresponding to the LOD.

	**S (nm·mL/ng)**	**o (nm)**	**σ (nm)**	**n #**	**R_{LOD} (nm)**	**LOD (ng/mL)**
**(i) Direct detection**						
**Blood plasma**	$8.13 \cdot 10^{-4}$	$3.40 \cdot 10^{-1}$	$1.18 \cdot 10^{-2}$	15	$3.07 \cdot 10^{-2}$	37.8
**Blood plasma + Ab_{IgG}**	$8.49 \cdot 10^{-4}$	$5.07 \cdot 10^{-2}$	$4.05 \cdot 10^{-3}$	15	$1.05 \cdot 10^{-2}$	12.4
**(ii) AuNP-enhanced detection**						
**Blood plasma**	$2.01 \cdot 10^{-1}$	71.8	$5.25 \cdot 10^{-2}$	13	$1.40 \cdot 10^{-1}$	0.694
**Blood plasma + Ab_{IgG}**	$2.98 \cdot 10^{-1}$	0.370	$2.53 \cdot 10^{-2}$	13	$6.71 \cdot 10^{-2}$	0.225

S5. References

1. Homola, J. and M. Piliarik, *Surface plasmon resonance (SPR) sensors*. 2006: Springer.
2. De Feijter, J., d.J. Benjamins, and F. Veer, *Ellipsometry as a tool to study the adsorption behavior of synthetic and biopolymers at the air–water interface.* Biopolymers: Original Research on Biomolecules, 1978. **17**(7): p. 1759-1772.
3. Tumolo, T., L. Angnes, and M.S. Baptista, *Determination of the refractive index increment (dn/dc) of molecule and macromolecule solutions by surface plasmon resonance.* Analytical Biochemistry, 2004. **333**(2): p. 273-279.
4. Hefta, L.J., M. Neumaier, and J.E. Shively, *Kinetic and affinity constants of epitope specific anti-carcinoembryonic antigen (CEA) monoclonal antibodies for CEA and engineered CEA domain constructs.* Immunotechnology, 1998. **4**(1): p. 49-57.
5. Bjerner, J., et al., *Protein epitopes in carcinoembryonic antigen: Report of the ISOBM TD8 Workshop.* Tumor Biology, 2002. **23**(4): p. 249-262.
6. Sun, H., et al., *Advances in carcinoembryonic antigen detection: a review of clinical applications and standardization.* Analytical and Bioanalytical Chemistry, 2025. **417**: p. 1751-1764.
7. Ermini, M.L., et al., *Peptide functionalization of gold nanoparticles for the detection of carcinoembryonic antigen in blood plasma via SPR-based biosensor.* Frontiers in Chemistry, 2019. **7**: p. 40.
8. Bastús, N.G., J. Comenge, and V. Puntes, *Kinetically controlled seeded growth synthesis of citrate-stabilized gold nanoparticles of up to 200 nm: size focusing versus Ostwald ripening.* Langmuir, 2011. **27**(17): p. 11098-11105.
9. Špringer, T., et al., *Functional gold nanoparticles for optical affinity biosensing.* Analytical and Bioanalytical Chemistry, 2017. **409**(16): p. 4087-4097.
10. Špringer, T., M. Piliarik, and J. Homola, *Surface plasmon resonance sensor with dispersionless microfluidics for direct detection of nucleic acids at the low femtomole level.* Sensors and Actuators B: Chemical, 2010. **145**(1): p. 588-591.
11. Špringer, T., M. Piliarik, and J. Homola, *Real-time monitoring of biomolecular interactions in blood plasma using a surface plasmon resonance biosensor.* Analytical and Bioanalytical Chemistry, 2010. **398**(5): p. 1955-61.
12. Rotmensch, S. and L.A. Cole, *False diagnosis and needless therapy of presumed malignant disease in women with false-positive human chorionic gonadotropin concentrations.* Lancet, 2000. **355**(9205): p. 712-5.
13. Zaitsu, K. and Y. Ohkura, *New fluorogenic substrates for horseradish peroxidase: rapid and sensitive assays for hydrogen peroxide and the peroxidase.* Analytical Biochemistry, 1980. **109**(1): p. 109-113.
14. Wen, F., et al., *Horseradish peroxidase functionalized fluorescent gold nanoclusters for hydrogen peroxide sensing.* Analytical Chemistry, 2011. **83**(4): p. 1193-1196.
15. Currie, L.A., *Nomenclature in evaluation of analytical methods including detection and quantification capabilities (IUPAC Recommendations 1995).* Pure and Applied Chemistry, 1995. **67**(10): p. 1699-1723.
